# Bayes Factors for Evaluating Latent Monotonicity in Polytomous Item Response Theory Models

**DOI:** 10.1007/s11336-019-09661-w

**Published:** 2019-02-21

**Authors:** Jesper Tijmstra, Maria Bolsinova

**Affiliations:** 1 Department of Methodology and Statistics, Faculty of Social Sciences, Tilburg University, PO Box 90153, 5000 LE Tilburg, The Netherlands; 2 ACTNext, Iowa City, USA

**Keywords:** Latent monotonicity, manifest monotonicity, item response theory, polytomous IRT, nonparametric IRT, Bayes factor

## Abstract

The assumption of latent monotonicity is made by all common parametric and nonparametric polytomous item response theory models and is crucial for establishing an ordinal level of measurement of the item score. Three forms of latent monotonicity can be distinguished: monotonicity of the cumulative probabilities, of the continuation ratios, and of the adjacent-category ratios. Observable consequences of these different forms of latent monotonicity are derived, and Bayes factor methods for testing these consequences are proposed. These methods allow for the quantification of the evidence both in favor and against the tested property. Both item-level and category-level Bayes factors are considered, and their performance is evaluated using a simulation study. The methods are applied to an empirical example consisting of a 10-item Likert scale to investigate whether a polytomous item scoring rule results in item scores that are of ordinal level measurement.

**Electronic supplementary material:**

The online version of this article (10.1007/s11336-019-09661-w) contains supplementary material, which is available to authorized users.

## Latent Monotonicity for Polytomously Scored Items

The property of latent monotonicity is one of the core assumptions in item response theory (IRT) models for both dichotomous and polytomous data. For IRT models for dichotomous data, the functioning of an item is captured by the item response function $\pi _{i}(\theta ) =P(X_{i} = 1|\theta )$, which describes the probability of obtaining a positive score (e.g., a correct response) for item *i* as a function of the latent variable $\theta$. Here, the assumption of latent monotonicity states that $\pi _{i}(\theta )$ is monotonically nondecreasing over $\theta$. As such, it captures the notion that on a well-functioning item persons of higher ability should never have a lower probability of providing a correct response than persons of lower ability. This makes it a statistical assumption that captures an important qualitative requirement for valid measurement, as a violation suggests that the item does not function adequately. It can also be seen as a check of the ordinality of measurement, in the sense that an item score of 1 is always indicative of higher ability than an item score of 0.

For polytomous items, one can likewise consider the question whether the item score is of ordinal level measurement. As in dichotomous IRT, all common polytomous IRT models assume some form of ordinality of the item score. This is captured by the assumption of latent monotonicity, which in polytomous IRT appears in various forms. In polytomous IRT, the item-category response function $\pi _{ij}(\theta ) = P(X_{i} = j|\theta )$ is not modeled directly, but rather particular functions of $\varvec{\pi }_i(\theta )=\{\pi _{i0}(\theta ),\ldots ,\pi _{im}(\theta )\}$ are used as the building block of the IRT model, where *m* is the highest possible realization of $X_{i}$. Three such possible building blocks are commonly considered: a cumulative probability (CP), a continuation ratio (CR), and an adjacent-category (AC) ratio.

For cumulative probability IRT models, the function

1 $$\begin{aligned} \xi _{ij}(\theta ) = \sum _{k=j}^{m} \pi _{ik}(\theta ) \end{aligned}$$

is considered for $j\in [1:m]$, which is the probability of obtaining score *j* or higher on item *i*. Common parametric IRT models that make use of this building block are the graded response model and its generalizations (Samejima, 1969). In each of these models, $\xi _{ij}(\theta )$ is assumed to be monotonically nondecreasing in $\theta$ for $j\in [1:m]$.^1^ Monotonicity of $\xi _{ij}(\theta )$ for $j\in [1:m]$ is also a defining assumption of the nonparametric graded response model (Hemker, Sijtsma, Molenaar, & Junker, 1996), the double monotonicity (Molenaar, 1997) and the strong double monotonicity model (Sijtsma & Hemker, 1998), and the ISOP model (Scheiblechner, 1995).

For continuation ratio IRT models, the function

2 $$\begin{aligned} \phi _{ij}(\theta ) = \frac{\sum _{k=j}^{m}\pi _{ik}(\theta )}{\sum _{k=j-1}^{m} \pi _{ik}(\theta )} \end{aligned}$$

is considered for $j\in [1:m]$, which is the probability of obtaining score *j* or higher given that at least a score of $j-1$ has been obtained. Common parametric IRT models that make use of this building block are the sequential models (Tutz, 1990). In these models, $\phi _{ij}(\theta )$ is assumed to be monotonically nondecreasing in $\theta$ for $j\in [1:m]$. Monotonicity of $\phi _{ij}(\theta )$ for $j\in [1:m]$ is also a defining assumption of the nonparametric sequential model (Hemker, van der Ark, & Sijtsma, 2001).

For adjacent-category IRT models, the function

3 $$\begin{aligned} \psi _{ij}(\theta ) = \frac{\pi _{ij}(\theta )}{\pi _{i(j-1)}(\theta )+\pi _{ij}(\theta )} \end{aligned}$$

is considered for $j\in [1:m]$, which is the probability of obtaining score *j* given that the score is either *j* or $j-1$. Common parametric IRT models that make use of this building block are the partial credit model and its generalizations (Masters, 1982; Muraki, 1992), and the rating scale model (Andrich, 1978). In each of these models, $\psi _{ij}(\theta )$ is assumed to be monotonically nondecreasing in $\theta$ for $j\in [1:m]$. Monotonicity of $\psi _{ij}(\theta )$ for $j\in [1:m]$ is also a defining assumption of the nonparametric partial credit model (Hemker, Sijtsma, Molenaar, & Junker, 1997).

Thus, in the context of polytomous IRT three forms of latent monotonicity are relevant for each item: monotonicity of $\xi _{ij}(\theta )$, $\phi _{ij}(\theta )$, and $\psi _{ij}(\theta )$ in $\theta$, for $j\in [1:m]$. Furthermore, these three properties are nested: If monotonicity of $\psi _{ij}(\theta )$ holds for $j\in [1:m]$, then monotonicity of $\phi _{ij}(\theta )$ also holds, and monotonicity of $\phi _{ij}(\theta )$ for $j\in [1:m]$ implies monotonicity of $\xi _{ij}(\theta )$ (Van der Ark, 2005). All three properties imply latent monotonicity of the expected item score, which has been studied by Ligtvoet and Vermunt (2012).

It may be noted that despite their differences in building blocks, all common parametric polytomous IRT models assume all three forms of monotonicity (Van der Ark, 2005). In this sense, testing the monotonicity of $\xi _{ij}(\theta )$, $\phi _{ij}(\theta )$, or $\psi _{ij}(\theta )$ all can be seen as a test of the possible applicability of parametric polytomous IRT models to the data, with a test for monotonicity of $\psi _{ij}(\theta )$ being the most stringent one due to the nestedness of the three properties.

Unlike the parametric IRT models, the nonparametric polytomous IRT models do differ in which forms of monotonicity are assumed (Van der Ark, 2001): The nonparametric graded response model only assumes monotonicity of all $\xi _{ij}(\theta )$, the nonparametric sequential model additionally assumes all $\phi _{ij}(\theta )$ to be nondecreasing over $\theta$, and the nonparametric adjacent-category model assumes all three forms of monotonicity. By not assuming all forms of monotonicity, the nonparametric graded response model and nonparametric sequential model are more flexible, but it should be kept in mind that since these models do not imply monotonicity of $\psi _{ij}(\theta )$ categories are not assumed to be ordered in the sense that the probability of choosing a higher category over a lower category does not necessarily increase monotonically over $\theta$. Depending on the purpose of the considered application, this might be an important limitation in practice. Generally, establishing which of the three forms of monotonicity hold can help one determine which nonparametric polytomous IRT models might be appropriate for modeling the data.

While most IRT tools for evaluating item functioning operate within a specific parametric framework, the question whether any of these three forms of monotonicity hold takes precedence over the choice of a particular IRT model. In this sense, checking the fit of an item to a particular IRT model does not directly address the question whether the item score is of ordinal measurement, as misfitting items might still be ordinal, and items with reasonable overall fit might still display local violations of the monotonicity of $\xi _{ij}(\theta )$, $\phi _{ij}(\theta )$, or $\psi _{ij}(\theta )$. Thus, standard IRT tools are not designed to address the question whether forms of latent monotonicity hold for an item.

As an alternative to parametric approaches, it has been suggested to use the nonparametric item-scalability coefficient *H* for the evaluation of items (Mokken, 1970; Sijtsma & Molenaar, 2002). The coefficient is based on the average covariance between the item score of the item that is considered and the scores on the other items in the test, and as such does not rely on any particular IRT model. If monotonicity holds, *H* will be nonnegative (Rosenbaum, 1984), and hence checking whether one can reject $H \ge 0$ constitutes a formal test for monotonicity. However, since there is no reason to expect *H* to be negative if monotonicity is violated, this test will in practice only constitute a minimal check for violations and may not be optimal for detecting such violations. If one alternatively decides to contrast *H* with a number larger than 0 (e.g., with .3, Sijtsma & Molenaar, 2002; Kuijpers, Van der Ark, & Croon, 2013), one no longer formally tests for monotonicity as *H* is only guaranteed to be nonnegative.

Given how crucial the assumption of latent monotonicity is for the validity of measurement, one would ideally like a method that evaluates this property to give the user information about how plausible that property is. That is, ideally one would like to obtain information about the extent to which the data support the notion that the property actually holds—rather than just informing the user that a statistical test failed to reject this hypothesis. Such a quantification of the evidence present in the data both in favor and against a hypothesis is provided by the Bayes factor  (BF; Jeffreys, 1935; Kass & Raftery, 1995). Unlike methods that rely on significance tests, BF methods allow for the conclusion that there is support in favor of the model assumption, and also quantify the strength of this support (Wagenmakers, 2007). In this way, they can be helpful to users who hope to validate their model assumptions (Tijmstra, 2018).

While for the evaluation of latent monotonicity in dichotomous IRT BF methods have been proposed (Tijmstra, Hoijtink, & Sijtsma, 2015), no such methods have been developed in the context of polytomous IRT. These methods for dichotomous IRT focus on testing whether the implications of latent monotonicity for manifest item score probabilities (i.e., manifest monotonicity) hold for a particular item. Since the BF methods for dichotomous IRT perform well both in terms of the true positive and true negative rate as well as the false positive and false negative rate,^2^ developing these methods for polytomous IRT holds the promise to improve the quality of the assessment of the assumptions of measurement for tests that produce polytomous data.

This paper proposes BF methods for evaluating latent monotonicity in polytomous IRT. Developing BF methods for polytomous response data is not a straightforward extension of the methods used for dichotomous response data, as several challenges are present in the polytomous case that are not present in the dichotomous case. First, in polytomous IRT latent monotonicity is present in three different forms, matching the three different building blocks that can be used. Methods for evaluating each of these forms of monotonicity need to considered, and their relative usefulness needs to be evaluated. Each of these properties deals with constraints that do not directly apply to the item-category functions, but rather apply at the level of the building blocks, and hence different constraints need to be imposed (and different conditional distributions need to be considered) than in the dichotomous case.

Second, the choice of the manifest score over which monotonicity is evaluated is not as straightforward as in dichotomous IRT. A proof is presented in this paper that shows that if the manifest score stochastically orders the latent variable and is conditionally independent of the item score, the manifest score can be used to evaluate latent monotonicity. However, as noted by Hemker, Sijtsma, Moleanaar, and Junker (1996), under most polytomous IRT models the sumscore is not guaranteed to stochastically order the latent variable, and hence using the restscore as the manifest score when evaluating latent monotonicity for a test with polytomous data is not advisable.

Third, since a polytomous item is characterized by multiple item-category functions, for each item latent monotonicity implies multiple sets of order constraints (one for each item-category function). This raises the question what the most optimal way is of evaluating latent monotonicity for an item, from both a statistical and a practical perspective. In this paper, both an item-level approach (evaluating all constraints imposed on an item using a single BF) and a category-level approach (evaluating the constraints using category-level BFs and combining these results) are proposed, their performance is compared, and the relative advantages and disadvantages of the two approaches are considered.

The structure of the paper is the following. In Sect. 2, observable consequences of the three forms of latent monotonicity are derived at the level of the manifest score, which results in three forms of manifest monotonicity that can be assessed. Section 3 presents item-level BF methods for evaluating each of the three forms of monotonicity. In Sect. 4, category-level BF methods are proposed as a possible alternative to the item-level approach of Sect. 3. Section 5 presents a simulation study, in which the performance of the item-level and the category-level methods is investigated by considering the proportion of replications in which the different methods suggest strong evidence in favor or against the three forms of latent monotonicity. Both a well-behaved item and two items that show a violation of latent monotonicity are considered as the focal item. Section 6 deals with an application of the procedures to empirical data, where the methods are used to study whether for a test consisting of 10 5-category Likert items one can conclude that the different forms of latent monotonicity hold. The paper concludes with a discussion.

## Observable Consequences of Latent Monotonicity

For notational convenience, the subscript *i* is dropped in the remainder of the manuscript, such that *X* refers to the score on the item for which monotonicity is evaluated. Both the item-category functions and the building blocks in Eqs. 1,  2, and  3 can be presented as conditional probabilities of the following form:

4 $$\begin{aligned} P(X \in S_{1}|X \in S_{2},\theta ), \end{aligned}$$

where $S_{1}$ and $S_{2}$ are sets of possible realizations of *X* and where $S_{1}$ is a subset of $S_{2}$. This formula is general in form and encompasses both $\pi _j(\theta )$ if $S_{1} = \{j \}$ and $S_{2} = \{0, \ldots , m \}$ as well as the three different building blocks for polytomous item response theory presented in Eqs. 1, 2, and 3. For example, $\phi _{j}(\theta )$ is obtained if one chooses $S_{1} = \{j, \ldots , m\}$ and $S_{2} = \{j - 1, j, \ldots , m\}$.

The three different forms of latent monotonicity discussed in the previous section all amount to assuming different conditional probabilities $P(X \in S_{1}|X \in S_{2},\theta )$ to be monotonically nondecreasing over $\theta$. Since $\theta$ is not observed, none of these forms of latent monotonicity can be tested directly. However, these latent properties may result in observable consequences at the level of manifest scores if certain conditions are met, which can be used to test whether the latent property is violated. That is, latent monotonicity may imply a form of manifest monotonicity (MM), for which statistical tests can be developed.

Let us consider a manifest score *Y* with realization $r = 0, \ldots , R$, which can be any composite score based on items in the test, such as for example the restscore. We can specify the manifest counterpart of the conditional probabilities: $P(X\in S_1|X\in S_2,Y=r)$. The property of MM for manifest score *Y* can be defined as

5 $$\begin{aligned} P(X\in S_1|X\in S_2,Y=r)\le P(X\in S_1|X\in S_2,Y=r+1), \quad \forall r\in [0:(R-1)]. \end{aligned}$$

While monotonicity of $P(X \in S_{1}|X \in S_{2},\theta )$ (i.e., latent monotonicity) is not a sufficient condition for MM (see for example the counterexample provided by Junker and Sijtsma in the context of dichotomous IRT; 2000), adding the condition that the manifest score stochastically orders the latent variable (SOL; Hemker et al., 1997) and assuming that *X* and *Y* are independent conditional on $\theta$ turns out to be sufficient for the translation of any form of monotonicity from the latent to the manifest level, as is stated in the following theorem:

### Theorem

If $P(X \in S_{1}|X \in S_{2},\theta )$ is a monotonically nondecreasing function of $\theta$, if the manifest score *Y* stochastically orders $\theta$, and if *X* and *Y* are independent conditional on $\theta$, MM as specified in Eq. 5 holds for *Y*.

### Proof

The conditional probability given the manifest score is equal to the following integral:

6 $$\begin{aligned} P(X\in S_1|X\in S_2,Y=r) = \int P(X \in S_{1}|X \in S_{2},\theta , Y=r)g(\theta |Y=r) {\hbox {d}}\theta , \end{aligned}$$

where $g(\theta \,|\, Y=r)$ is the conditional distribution of the latent variable given the manifest score. The property of conditional independence of *X* and *Y* given $\theta$ allows one to further rewrite it as

7 $$\begin{aligned} \int P(X \in S_{1}|X \in S_{2},\theta , Y=r)g(\theta |Y=r) {\hbox {d}}\theta =\int P(X \in S_{1}|X \in S_{2},\theta )g(\theta |Y=r) {\hbox {d}}\theta , \end{aligned}$$

where the righthand side is the expected value of $P(X \in S_{1}|X \in S_{2},\theta )$ given that the manifest score equals *r*.

The property of SOL states that the conditional distributions of $\theta$ given the increasing values of the manifest score *Y* are stochastically ordered:

8 $$\begin{aligned} g(\theta \,|\,Y=r)\le _{st} g(\theta \,|\, Y=r+1), \quad \forall r\in [0:(R-1)]. \end{aligned}$$

Since $P(X \in S_{1}|X \in S_{2},\theta )$ is a nondecreasing function of $\theta$ and the conditional distributions in Eq. 8 are stochastically ordered, the following inequality holds (Shaked & Shanthikumar, 2007):

9 $$\begin{aligned}&\int P(X \in S_{1}|X \in S_{2},\theta )g(\theta |Y=r) {\hbox {d}}\theta \nonumber \\&\quad \le \int P(X \in S_{1}|X \in S_{2},\theta )g(\theta |Y=r+1) {\hbox {d}}\theta , \forall r\in [0:(R-1)], \end{aligned}$$

which is equivalent to MM in Eq. 5. This concludes the proof. $\square$

Thus, if a manifest score is considered for which SOL holds and which is conditionally independent of *X*, the three forms of latent monotonicity imply monotonic order constraints for their manifest counterparts: (1) MM for cumulative probabilities:

10 $$\begin{aligned} \xi _{j0} \le \xi _{j1} \le \cdots \le \xi _{jR}, \quad \forall {j\in [1:m]}, \end{aligned}$$

where

11 $$\begin{aligned} \xi _{jr} = \sum _{k = j}^{m}\pi _{jr}, \end{aligned}$$

where $\pi _{jr} = P(X = j|Y = r)$; (2) MM for continuation ratios:

12 $$\begin{aligned} \phi _{j0} \le \phi _{j1} \le \ldots \le \phi _{jR}, \quad \forall {j\in [1:m]}, \end{aligned}$$

where

13 $$\begin{aligned} \phi _{jr} = \frac{\sum _{k = j}^{m}\pi _{kr}}{\sum _{k = j-1}^{m}\pi _{kr}}; \end{aligned}$$

(3) MM for adjacent-category ratios:

14 $$\begin{aligned} \psi _{j0} \le \psi _{j1} \le \ldots \le \psi _{jR}, \quad \forall {j\in [1:m]}, \end{aligned}$$

where

15 $$\begin{aligned} \psi _{jr} = \frac{\pi _{jr}}{\pi _{jr} + \pi _{(j-1)r}}. \end{aligned}$$

Equations 10, 12, and 14 each imply *m* sets of *R* order constraints that can be tested based on the observed proportions conditional on the manifest score.

The formulations in Eqs. 10, 12, and 14 leave it open which particular manifest score is considered. However, since the translation of latent monotonicity to the manifest level relied upon a manifest score being considered for which SOL holds and which is conditionally independent of the item score, this places restrictions on which manifest score should be considered. Firstly, using the total score (or any manifest score that includes the score on the item that is considered) may not be recommended, as this likely results in a violation of conditional independence of *X* and *Y*. Secondly, it may also not be advisable to work with composite scores that consist of polytomous item scores, as the property of SOL is not implied for the sumscore under most polytomous IRT models (Hemker et al., 1996). Since SOL is implied by almost all IRT models for sumscores based on *dichotomous* item scores, the preferable option would be to make use of a composite score consisting of dichotomous item scores, which on a test with polytomous items can be realized by making use of dichotomized item scores (e.g., by using a median split for each polytomous item). However, it should be noted that working with dichotomized item scores in itself does not guarantee SOL to hold, but rather still requires the construction of a manifest score based on a set of items that are generally well behaved. If many items on the test are of questionable quality, it may not be plausible to assume that SOL holds for the restscore (regardless of whether dichotomized scores are used), and hence the decision which item scores to include in the manifest score should be made with care.

## Item-Level Bayes Factors for Manifest Monotonicity

Equations 10, 12, and 14 each specify a form of MM. The hypothesis that MM of type *z* holds for the item that is considered corresponds to

16 $$\begin{aligned} H_{z}: \quad z_{j(r-1)}\le z_{jr}, \quad \forall r\in [1:R],\forall j\in [1:m], \end{aligned}$$

where *z* can be $\xi$, $\phi$, or $\psi$. $H_{z}$ can be contrasted with its negation, denoted by $\lnot H_{z}$, which is the hypothesis that at least one of the constraints in 16 is violated. The hypotheses $H_{z}$ and $\lnot H_{z}$ are mutually exclusive and together are exhaustive; therefore, MM can be evaluated by contrasting these two hypotheses.

The relative support for two competing hypotheses can be quantified using BFs (Jeffreys, 1935; Kass & Raftery, 1995). The BF balances the fit of the two hypotheses against their complexity and provides a continuous measure of the extent to which the data favor one hypothesis over the other. A BF does not necessarily force a dichotomous decision (accept or reject) on the user, but one can still opt to make use of decision rules based on the amount of evidence that one considers to be sufficient for accepting or rejecting a hypothesis (see e.g., Tijmstra et al., 2015).

Following the framework proposed by Hoijtink (2011), the complexity and the fit of an inequality constrained hypothesis such as (16) can be defined as the proportions of the prior and posterior distribution of the parameters of interest that is in accordance with this hypothesis, respectively. The BF for testing MM of type *z* can be computed as:

17 $$\begin{aligned} {\hbox {BF}}_z=\frac{f_z(1-c_z)}{c_z(1-f_z)}, \end{aligned}$$

where $c_z$ and $f_z$ are the complexity and the fit of $H_{z}$, and $(1-c_z)$ and $(1-f_z)$ are the complexity and the fit of $\lnot H_{z}$. ${\hbox {BF}}_z$ provides a continuous measure of the degree to which the data favor $H_z$ over $\lnot H_{z}$. On the log-scale, a value smaller than $-3$ or larger than 3 is often considered to constitute ‘strong evidence’ for one hypothesis over the other (Kass & Raftery, 1995). In line with this, we propose the following rule for categorizing the evidence in the data: $H_z$ is considered to be supported if $\ln ({\hbox {BF}}_z) \ge 3$, $\lnot H_{z}$ is considered to be supported if $\ln ({\hbox {BF}}_z) \le -3$, and the evidence is considered inconclusive if $-3< \ln ({\hbox {BF}}_z) < 3$.

### Prior Distribution, Likelihood Function, and Posterior Distribution

The hypotheses $H_{z}$ and $\lnot H_{z}$ impose constraints on $\varvec{\pi },$ the $(m + 1) \times (R + 1)$ matrix with elements $\pi _{jr}$, and where $\varvec{\pi }_{\varvec{\cdot } r}$ refers to the *r*-th column in the matrix. To evaluate the fit and the complexity of these two hypotheses, a prior and posterior distribution of $\varvec{\pi }$ need to be specified. In order to ensure that for every $j\in [1:m]$ any ordering of $z_{j0}\ldots ,z_{jR}$ is equally likely a priori, one can specify the prior distribution to be the following:

18 $$\begin{aligned} f(\varvec{\pi })=\prod _{r=0}^R \text {Dirichlet}(\varvec{\pi }_{\varvec{\cdot } r}; \mathbf {1}_{m+1}). \end{aligned}$$

Assuming the scores of the item to have a multinomial distribution for each value of the manifest score, the likelihood of the data is:

19 $$\begin{aligned} L(\varvec{\pi };\mathbf {X})=\prod _{r=0}^R\prod _{j=0}^m \pi _{jr}^{N_{jr}} , \end{aligned}$$

where $N_{jr}$ is the number of persons with the item score of *j* and the manifest score of *r*. The Dirichlet distribution is a conjugate prior for the multinomial model; therefore, the posterior of each $\varvec{\pi }_{\varvec{\cdot } r}$ is also a Dirichlet distribution:

20 $$\begin{aligned} f(\varvec{\pi }\,|\, \mathbf {X})=\prod _{r=0}^R \text {Dirichlet}(\varvec{\pi }_{\varvec{\cdot } r};1+N_{0r},1+N_{1r},\ldots ,1+N_{mr}). \end{aligned}$$

Since the elements of each $\varvec{\pi }_{\varvec{\varvec{\cdot }} r}$ are not independent of each other, it is more convenient to reparameterize the model in terms of $\varvec{\phi }$ (i.e., the *m* by $R+1$ matrix containing all $\phi _{jr}$s, the manifest continuation ratios), the elements of which are independent of each other. The prior and posterior of $\varvec{\pi }$ translate to the following prior and posterior of $\varvec{\phi }$:

21 $$\begin{aligned} f(\varvec{\phi })= & {} \prod _{r=1}^R\prod _{j=1}^{m} \mathcal {B}(\phi _{jr}; 1+(m-j),1); \end{aligned}$$

22 $$\begin{aligned} f(\varvec{\phi }\,|\, \mathbf {X})= & {} \prod _{r=1}^R\prod _{j=1}^{m} \mathcal {B}\left( \phi _{jr}; 1+(m-j)+\sum _{k=j}^{m}N_{kr},1+N_{(j-1)r}\right) . \end{aligned}$$

Here, we also show how $\varvec{\pi }$, $\varvec{\xi }$ (i.e., the *m* by $R+1$ matrix containing all $\xi _{jr}$s), and $\varvec{\psi }$ (i.e., the *m* by $R+1$ matrix containing all $\psi _{jr}$s) can be presented as a function of $\varvec{\phi }$. The most simple relationship is between $\varvec{\xi }$ (the cumulative probability matrix) and $\varvec{\phi }$, since $\xi _{jr}$ is equal to the numerator of $\phi _{jr}$ (see Eqs. 11 and 13). We have:

23 $$\begin{aligned} \xi _{jr}=\prod _{k=1}^j \phi _{kr}, \forall j\in [1:m], \quad \forall r\in [0:R]. \end{aligned}$$

This can be used to derive the relationship between $\varvec{\pi }$ and $\varvec{\phi }$, since the elements of $\varvec{\pi }_{\varvec{\cdot } r}$ can be presented as the difference between neighboring $\xi _{jr}$s:

24 $$\begin{aligned} \pi _{jr}=\xi _{jr}-\xi _{(j+1)r}=\prod _{k=1}^j \phi _{kr}-\prod _{k=1}^{j+1} \phi _{kr},\forall j\in [1:m], \quad \forall r\in [0:R], \end{aligned}$$

where $\xi _{(m+1)r}\equiv \phi _{(m+1)r}\equiv 0,\forall r\in [0:R].$^3^ Finally, using the definition of $\psi _{jr}$ (see Eq. 15), we obtain:

25 $$\begin{aligned} \psi _{jr}=\frac{\prod \nolimits _{k=1}^j \phi _{kr}-\prod \nolimits _{k=1}^{j+1} \phi _{kr}}{\prod \nolimits _{k=1}^j \phi _{kr}-\prod \nolimits _{k=1}^{j+1} \phi _{kr}+\prod \nolimits _{k=1}^{j-1} \phi _{kr}-\prod \nolimits _{k=1}^{j} \phi _{kr}}=\frac{\phi _{jr}-\phi _{jr}\phi _{(j+1)r}}{1-\phi _{jr}\phi _{(j+1)r}}, \quad \forall j\in [1:m], \forall r\in [0:R], \end{aligned}$$

which is derived by dividing both the numerator and the denominator by $\prod \nolimits _{k=1}^{j-1}\phi _{kr}$.

### Estimating the Fit of a Hypothesis

Let us by $Q_{zr}$ denote a set of constraints $z_{j(r-1)}\le z_{jr}, \forall j\in [1,m]$. The fit of $H_{z}$ corresponds to the proportion of draws from the posterior distribution of $\varvec{\phi }$ in which the constraints $Q_{z1},\ldots ,Q_{zR}$ jointly hold, denoted by $g(Q_{z1}\ldots ,Q_{zR})$. $g(Q_{z1}\ldots ,Q_{zR})$ can in principle be estimated by sampling from the unconstrained posterior distribution of $\varvec{\phi }$ in Eq. 22. However, since this proportion is usually very small, a very large number of samples might be needed to accurately estimate $g(Q_{z1}\ldots ,Q_{zR})$. It is computationally more efficient (Mulder et al., 2009) to estimate this proportion by using the following decomposition:

26 $$\begin{aligned} g(Q_{z1},Q_{z2},\ldots ,Q_{zR})=g(Q_{z1})g(Q_{z2}|Q_{z1})g(Q_{z3}| Q_{z1},Q_{z2})\times \ldots \times g(Q_{zR}| Q_{z1},Q_{z2},\ldots ,Q_{z(R-1)}). \nonumber \\ \end{aligned}$$

$g(Q_{z1})$ can be estimated by sampling from the unconstrained posterior of $\varvec{\phi }_{\varvec{\cdot } 0}$ and $\varvec{\phi }_{\varvec{\cdot } 1}$:

27 $$\begin{aligned}&f(\varvec{\phi }_{\varvec{\cdot } 0},\varvec{\phi }_{\varvec{\cdot } 1}\,|\, \mathbf {X})=\prod _{j=1}^{m} \mathcal {B}\left( \phi _{j0}; 1+(m-j)+\sum _{k=j}^{m}N_{k0},1+N_{(j-1)0}\right) \nonumber \\&\quad \mathcal {B}\left( \phi _{j1}; 1+(m-j)+\sum _{k=j}^{m}N_{k1},1+N_{(j-1)1}\right) \end{aligned}$$

and computing the proportion of samples in which the constraints $Q_{z1}$ hold. All subsequent components of Eq. 26 can be estimated by sampling from constrained posteriors: For each $s\in [1:(R-1)]$, one needs to sample from the constrained posterior $f(\varvec{\phi }_{\varvec{\cdot } 0},\ldots ,\varvec{\phi }_{\varvec{\cdot }(s+1)}\,|\,\mathbf {X}, Q_{z1},\ldots ,Q_{zs}),$ and compute the proportion of samples in which the constraints $Q_{z(s+1)}$ hold to obtain an estimate of $g(Q_{z(s+1)}\,|\, Q_{z1},\ldots ,Q_{zs})$.

The constrained posterior of $\varvec{\phi }_{\varvec{\cdot } 0},\ldots ,\varvec{\phi }_{\varvec{\cdot } (s+1)}$ given that the first *s* constraints hold is:

28 $$\begin{aligned}&f(\varvec{\phi }_{\varvec{\cdot } 0},\ldots ,\varvec{\phi }_{\varvec{\cdot } (s+1)}\,|\, \mathbf {X},Q_{z1},\ldots ,Q_{zs})=\prod _{j=1}^m \mathcal {B}\left( \phi _{j(s+1)}; 1+(m-j)+\sum _{k=j}^mN_{k(s+1)},1+N_{(j-1)(s+1)}\right. \nonumber \\&\quad \left. \times \prod _{r=1}^s \mathcal {B}(\phi _{jr}; 1+(m-j)+\sum _{k=j}^{m}N_{kr},1+N_{(j-1)r}\right) \mathcal {I}(a_{zjrs}\le \phi _{jr}\le b_{zjrs}), \end{aligned}$$

where $a_{zjrs}$ and $b_{zjrs}$ are the lower and upper bounds for $\phi _{jr}$ when the first *s* constraints of type *z* are considered. The exact specification of the lower and the upper bounds for the $\phi _{jr}$s for the three different types of MM is given in “Appendix A.”

While it is in principle possible to sample from the joint constrained posterior distribution in Eq. 28 directly using rejection sampling, this would be not computationally efficient. Therefore, we use a Gibbs sampler (Casella & George, 1992; Geman & Geman, 1984) to sample from this distribution, see “Appendix B” for the steps of the Gibbs sampler. After running the Gibbs sampler for a large number of iterations and discarding the burn-in, one needs to compute the proportion of samples for which $Q_{z(s+1)}$ holds, which gives an estimate of $g(Q_{z(s+1)}\,|\, Q_{z1},\ldots ,Q_{zs})$. By first sampling independently from Eq. 27 to estimate $g(Q_{z1})$ and then running $R-1$ Gibbs samplers to estimate the remaining components in Eq. 26, one can obtain an estimate of $g(Q_{z1},Q_{z2},\ldots ,Q_{zR})$.

### Estimating the Complexity of Manifest Monotonicity

For MM of the continuation ratios, the complexity can be derived analytically. Under the prior distribution in (18), each of the $(R+1)!$ possible orderings of $\{\phi _{j0},\ldots ,\phi _{jR}\}$ is equally likely. Since there are *m* such sets and since the continuation ratios for different item scores are independent, the complexity of $H_{\phi }$ is one divided by the total number of possible orderings, $((R+1)!)^m$.

The complexity of the MM of adjacent-category ratios and of the cumulative probabilities is not easily determined analytically, since for these types of monotonicity the constraints $\{z_{j0}\le \ldots \le z_{jR}\}$ and $z_{(j+1)0}\le \ldots \le z_{(j+1)R}$ are not independent of each other for $j\in [1:(m-1)]$. The complexity of these types of monotonicity can be determined using a simulation in which the complexity is estimated using the same Gibbs sampler as for estimating the fit but given no observations, that is $N_{jr}=0,\forall j\in [0:m],r\in [0:R]$.

## Category-Level Bayes Factors for Manifest Monotonicity

As Eqs. 10, 12 and 14 show, each form of MM consists of a set of order constraints for all categories from 1 to *m*, and each of these category-level sets of order constraints needs to hold for MM to hold for a polytomous item. Thus far, we have considered evaluating MM at the item-level, by considering the full set of constraints placed upon the matrix $\varvec{\phi }$. There may, however, be statistical or substantive reasons for evaluating the constraints placed upon $\varvec{\phi }$ row by row, rather than all at once. This would amount to considering for each $j = 1, \ldots , m$ whether its order constraints hold, which corresponds to evaluating MM at the level of the categories rather than at the item-level.

The item-level approach considers all constraints placed upon $\varvec{\phi }$ together, resulting in one overall measure of support (${\hbox {BF}}_z$). However, because the BF considers the overall fit of the hypothesis, evidence in favor of some of the constraints may ‘overwhelm’ evidence against some of the other constraints (Tijmstra et al., 2015). This means that if there is an issue with one particular response category of the item (resulting in a violation of monotonicity) while the other categories behave normally, being able to reliably detect this violation may require a larger sample size than would have been needed if one had specifically considered the order constraints at the category level. Thus, if one considers it plausible that violations of monotonicity may be category-specific, considering monotonicity at the category level rather than at the item level may result in a higher power to detect these violations.

In addition to considerations of power, there may also be substantive reasons to specifically consider the functioning of each of the response categories on the item. Each response option may be qualitatively different, and it may be relevant to investigate whether each of these options functions as intended. For example, one might suspect that on a seven-point Likert item respondents do not meaningfully distinguish two adjacent response options (e.g., ‘somewhat agree’ and ‘agree’). This substantive hypothesis could translate to the expectation that monotonicity does not hold for these particular categories, which might be best investigated using category-level tests for MM rather than item-level tests. Such tests may for example help decide whether certain item scores should be merged before running IRT analyses, and whether for subsequent test administrations the number of response options presented to respondents should be reduced.

To compute the BF for category-level MM of type *z* for a category *j* (${\hbox {BF}}_{zj}$), one needs to evaluate the fit and complexity of the hypotheses of interest (i.e., $H_{zj}$ and $\lnot H_{zj}$). For all three types of MM the complexity of category *j* is equal to $\frac{1}{(R+1)!}$ since of all $(R + 1)!$ possible orderings of $\{z_{j0},\ldots ,z_{jR}\}$ only 1 is in agreement with the hypothesis.

The fit of category-level MM can be estimated similarly to the fit of the item-level MM. Let us by $Q_{zjr}$ denote a set of constraints $z_{j(r-1)}\le z_{jr}$. The fit of the category-level MM can be estimated by:

29 $$\begin{aligned} g(Q_{zj1},\ldots ,Q_{zjR})=g(Q_{zj1})g(Q_{zj2}\,|\,Q_{zj1})\times \ldots \times g(Q_{zjR}\,|\,Q_{zj1},\ldots ,Q_{zj(R-1)}). \end{aligned}$$

To estimate this product, one needs to 1) sample $(z_{j0},z_{j1})$ from their unconstrained posterior, 2) for each $s\in [1:(R-1)]$ use a Gibbs sampler analogous to the one for evaluating the fit of item-level MM to sample from

30 $$\begin{aligned} f(z_{j0},\ldots ,z_{jr}\,|\, \mathbf {X},Q_{zj1},\ldots ,Q_{zjs}) \end{aligned}$$

and compute the proportion of samples in which the constraint $Q_{zj(s+1)}$ holds (see “Appendix C” for details).

The unconstrained posterior distribution of $z_{j0},\ldots ,z_{jR}$ can be derived from the posterior of $\varvec{\pi }$ and is

31 $$\begin{aligned} f(z_{j0},\ldots ,z_{jR}\,|\, \mathbf {X})=\prod _{r=0}^R\mathcal {B}(z_{jr};N_{zjr},S_{zjr}), \end{aligned}$$

where $N_{zjr}$ and $S_{zjr}$ depend on the type of MM: For AC, they are equal to $(N_{jr}+1)$ and $(N_{(j-1)r}+1)$; for CR, they are $(m-j+1+\sum _{k=j}^mN_kr)$ and $(1+N_{(j-1)r})$; and for CP, they are equal to $(m-j+1+\sum _{k=j}^m N_{kr})$ and $(j+\sum _{k=0}^{j-1}N_{kr})$.

## Simulation Study

The procedures described in Sects. 3 and 4 were implemented in R (R Core Team, 2016). Both the item-level and category-level BF should be able to capture evidence in favor and against latent monotonicity. To evaluate under which conditions they reliably point in the right direction, a simulation study was conducted to investigate their behavior under varying conditions, and to compare their performance to that of the item-scalability index *H*.

### Method

Four design factors were varied in the simulation study: (1) quality of the manifest score ($\alpha$; with the nonfocal items either having a low- or a high-quality discrimination parameter, as discussed below); (2) number of items in the manifest score ($R = 5, 10, 20$); (3) sample size ($N = 200, 500, 1000$); (4) number of categories on each item ($m = 2, 4$). A full factorial $2\times 3\times 3 \times 2$ design was used.

For the simulation study separately for the conditions with $m=2$ and for the conditions with $m=4$, 100 data sets with responses of 1000 persons to 40 monotone nonfocal items and 3 focal items were generated. In each data set, the person parameters were sampled from $\mathcal {N}(0,1)$. The responses to the nonfocal items were generated using the generalized partial credit model (GPCM; Muraki,^1992^):

32 $$\begin{aligned} \Pr (X_{i}=k\,|\,\theta ,\varvec{\delta }_i,\alpha _i)= \frac{\exp \left( \sum _{j=0}^k \alpha _i(\theta -\delta _{ij})\right) }{ \sum _{s=0}^{m}\exp \left( \sum _{j=0}^s \alpha _i( \theta -\delta _{ij})\right) }, \text {for } k = 0, \cdots , m. \end{aligned}$$

Here, the threshold parameters of every five consecutive items were $\varvec{\delta }_{\cdot 0}=\mathbf {0}$ and $\varvec{\delta }_{\cdot j}=\beta _j+\bar{\varvec{\delta }}$ for $j\in [1:m]$, where $\bar{\varvec{\delta }}= [-1,-0.5,0,0.5,1]$, and $\varvec{\beta }$ was equal to $[-1,1]$ for $m=2$ and $[-1,-\frac{1}{3},\frac{1}{3},1]$ for $m=4$. The slope parameters of the first 20 nonfocal items were equal to 0.75 (low discrimination condition), and the slope parameters of the last 20 nonfocal items were equal to 1.5 (high-discrimination condition).

Depending on the condition different parts of the replicated data sets were used. In the conditions with nonfocal items with low discrimination for each generated dataset, the responses of the first *N* persons to the items 1 through *R* were used. In the high-discrimination conditions, the responses of the first *N* persons to the items from 21 to $(20+R)$ from each generated data set were used. As the manifest score, the sumscore based on the dichotomized item scores on these nonfocal items was used, where the dichotomization was chosen such that the proportion of the responses above and below the boundary were as close to .5 as possible.

The three focal items were: (1) a monotone item, (2) an unfolding item, and (3) an item with switched response categories. The latter two items were chosen such that all three forms of latent monotonicity are violated, while differing in the type of violation. The response to the focal monotone item was generated under the GPCM with the threshold parameters equal to $$[0,-1,1]$$ for $$m=2$$ and $$[0,-1,-\frac{1}{3},\frac{1}{3},1]$$ for $$m=4$$, and a slope parameter of 1.5. To generate responses to the unfolding item, a pseudo-response $$X^{*}$$ with $$2m+1$$ categories was generated under the GPCM with the slope equal to 1.5 and threshold parameters $$\varvec{\delta }^*$$, where $$\delta _0^*=0$$ and the rest of the thresholds were set to be 2*m* equally spaced values between $$-2$$ and 2. The response to the focal item was then obtained by setting $$X=\min \{X^*,2m-X^*\}$$. To generate the responses to the item with switched categories, a pseudo-response $$X^{**}$$ with $$m+1$$ categories was generated under the GPCM with the same parameter values as for the monotone focal item. The response to the focal item was then obtained by re-coding the pseudo-response as follows: $$X=X^{**}$$ if $$X^{**}<(m-1)$$, and $$X=2m-X^{**}-1$$ if $$X^{**}\in [m-1,m]$$; that is, the order of the last two categories was switched.

For each of the focal items, the three forms of monotonicity were evaluated using both item- and category-level BFs, which were estimated using the procedures described in the previous two sections. The marginal probability of the first constraint holding was estimated using 100,000 draws from the posterior, and the conditional probabilities of each of the following $R-1$ constraints given all previous constraints were estimated using Gibbs samplers with 200,000 iterations each, of which the first 100,000 were discarded as burn-in. Since the BF is estimated to be 0 (i.e., infinitely strong evidence against monotonicity) if for any of the considered constraints 0 of the 100,000 draws are in line with that constraint (see Eqs. 26 and 29), a routine was implemented that checked whether if the next draw would have been a hit $\ln ({\hbox {BF}})$ would exceed $-3$ (i.e., whether obtaining a hit would change the qualification of the evidence). If this was the case, additional draws from that conditional posterior distribution were obtained with increments of 100,000, up to the point where some draws were in line with the constraint, or where obtaining a hit would not result in $\ln ({\hbox {BF}}) > -3$, or until 5 million draws had been obtained. In addition to the BFs, in each generated dataset for each focal item the scalability coefficient *H* was computed using the R package ‘mokken’ (Van der Ark, 2007, 2012).

The following outcome variables were used: (1) for each type of item-level BFs, the percentage of replications with $\ln ({\hbox {BF}}) \ge 3$ and the percentage of replications with $\ln ({\hbox {BF}}) \le -3$; (2) for each type of category-level BFs, the percentage of replications where $\ln ({\hbox {BF}}) \ge 3$ for **all** categories and the percentage of replications where $\ln ({\hbox {BF}}) \le -3$ for **at least** one of the categories; (3) the percentage of replications where the lower bound of the *H*-coefficient falls above .3 (i.e., significantly higher than .3, based on the 95% confidence interval) and the percentage of replications where the upper bound of the *H*-coefficient falls below .3 (i.e., significantly lower than .3, based on the 95% confidence interval).

### Results

Table 1 displays the results that were obtained for the well-behaved focal item (i.e., the ‘monotone item’). For the three-category item, there does not seem to be an issue with any of the measures with respect to mistakenly concluding the monotone item to be problematic: Only in a few conditions did some of the measures suggest evidence against monotonicity. For the BF measures as well as the *H*-coefficient, this happened in at most 1% of cases. Thus, none of the measures appear to have a high risk of inaccurately flagging a well-functioning three-category item as problematic. For the five-category item, the proportion of incorrectly flagged items is also low, with the exception of conditions with a small sample size ($N = 200$) and a large test length ($K = 20$), where especially the item-level BF shows an undesirable proportion of incorrect flags. In these conditions, sparsity appears to be an issue, since the procedure has to evaluate a table with 105 cells (5 by 21) over which only 200 persons are distributed. Once larger sample sizes are considered ($N = 500$ for the category-level approach and $N = 1000$ for the item-level approach), the proportion of false flags also becomes acceptable for these conditions.

**Table 1:** Results for the monotone focal item, displaying the percentage of replications in which support was found against monotonicity (outside of parentheses) and in favor of monotonicity (inside parentheses) for each of the seven measures, based on 100 replications.

*m*	$\alpha$	*R*	*N*	BF item level						BF category level							
				CP		CR		AC		CP		CR		AC		H	
2	0.75	5	200	0	(97)	0	(100)	0	(100)	0	(81)	0	(68)	0	(54)	0	(12)
			500	0	(100)	0	(100)	0	(100)	0	(99)	0	(96)	0	(90)	1	(24)
			1000	0	(100)	0	(100)	0	(100)	0	(100)	0	(99)	0	(98)	0	(25)
		10	200	0	(100)	0	(100)	0	(100)	0	(98)	0	(95)	0	(88)	0	(18)
			500	0	(100)	0	(100)	0	(100)	0	(100)	0	(100)	0	(100)	0	(26)
			1000	0	(100)	0	(100)	0	(100)	0	(100)	0	(100)	0	(100)	0	(42)
		20	200	0	(99)	0	(100)	0	(100)	0	(98)	1	(96)	1	(94)	0	(18)
			500	0	(100)	0	(100)	0	(100)	0	(100)	0	(100)	0	(100)	0	(33)
			1000	0	(100)	0	(100)	0	(100)	0	(100)	0	(100)	0	(100)	0	(46)
	1.5	5	200	0	(100)	0	(100)	0	(100)	0	(94)	0	(82)	0	(68)	0	(99)
			500	0	(100)	0	(100)	0	(100)	0	(100)	0	(99)	0	(97)	0	(100)
			1000	0	(100)	0	(100)	0	(100)	0	(100)	0	(100)	0	(100)	0	(100)
		10	200	0	(100)	0	(100)	0	(100)	0	(99)	0	(98)	0	(88)	0	(98)
			500	0	(100)	0	(100)	0	(100)	0	(100)	0	(100)	0	(100)	0	(100)
			1000	0	(100)	0	(100)	0	(100)	0	(100)	0	(100)	0	(100)	0	(100)
		20	200	0	(100)	0	(100)	0	(100)	0	(100)	0	(98)	0	(98)	0	(99)
			500	0	(100)	0	(100)	0	(100)	0	(100)	0	(100)	0	(100)	0	(100)
			1000	0	(100)	0	(100)	0	(100)	0	(100)	0	(100)	0	(100)	0	(100)
4	0.75	5	200	0	(100)	0	(100)	0	(100)	0	(93)	0	(16)	1	(1)	0	(100)
			500	0	(100)	0	(100)	0	(100)	0	(100)	0	(67)	0	(29)	0	(100)
			1000	0	(100)	0	(100)	0	(100)	0	(100)	0	(95)	0	(76)	0	(100)
		10	200	0	(100)	0	(100)	0	(100)	0	(100)	0	(56)	0	(15)	0	(100)
			500	0	(100)	0	(100)	0	(100)	0	(100)	0	(94)	0	(81)	0	(100)
			1000	0	(100)	0	(100)	0	(100)	0	(100)	0	(97)	0	(95)	0	(100)
		20	200	0	(100)	0	(100)	15	(85)	0	(100)	1	(73)	3	(34)	0	(100)
			500	0	(100)	0	(100)	4	(96)	0	(100)	0	(97)	0	(91)	0	(100)
			1000	0	(100)	0	(100)	1	(99)	0	(100)	0	(100)	0	(100)	0	(100)
	1.5	5	200	0	(100)	0	(100)	0	(100)	0	(89)	1	(16)	2	(0)	0	(100)
			500	0	(100)	0	(100)	0	(100)	0	(99)	0	(66)	0	(29)	0	(100)
			1000	0	(100)	0	(100)	0	(100)	0	(100)	0	(89)	0	(70)	0	(100)
		10	200	0	(100)	0	(100)	1	(99)	0	(100)	2	(44)	6	(10)	0	(100)
			500	0	(100)	0	(100)	0	(100)	0	(100)	0	(80)	1	(44)	0	(100)
			1000	0	(100)	0	(100)	0	(100)	0	(100)	0	(98)	1	(92)	0	(100)
		20	200	0	(100)	8	(92)	17	(83)	0	(100)	3	(48)	8	(8)	0	(100)
			500	0	(100)	0	(100)	13	(87)	0	(100)	0	(89)	3	(51)	0	(100)
			1000	0	(100)	0	(100)	1	(99)	0	(100)	0	(98)	0	(89)	0	(100)

Table 1 also shows for each condition the percentage of cases where the different measures found support for monotonicity (numbers in parentheses). These results show that the item-level BF more easily finds evidence that the item behaves monotonically than the category-level BF approach, where it is only concluded that support is found if *all* category-level $\ln ({\hbox {BF}})\hbox {s}$ exceed 3. The item-level BF approach finds support for all three forms of monotonicity in almost all cases. For the three-category item, in each condition at least 97% of cases support was found, while for the five-category item in all conditions at least 83% of cases support was found, with this percentage increasing with sample size. Due to the difference in the number of BFs that need to exceed the specified threshold of $\ln ({\hbox {BF}}) > 3$, the category-level approach more easily finds support for monotonicity for the three-category item than for the five-category item. However, for both types of items the percentage of cases in which support was found increases quickly with sample size, where for the three-category item and $N = 1000$ in each condition at least 98% of cases showed support for monotonicity, and in at least 70% of cases for the five-category item.

The results suggest that for both the item-level and the category-level approach, finding support for monotonicity of $\varvec{\xi }$ (i.e., cumulative probabilities) is easier than that of $\varvec{\phi }$ (i.e., continuation ratios), with finding support for monotonicity of $\varvec{\psi }$ (i.e., adjacent categories) being the most difficult. Especially for the five-category item, a large sample size ($N = 1000$) appears to be needed in most conditions to reliably find support for monotonicity of $\varvec{\psi }$ using the category-level approach. In contrast, for the *H*-coefficient the probability of findings support appears to depend mainly on the quality of the manifest score and the number of categories, where for the three-category item in all conditions with a weak manifest score support was found in less than 46% of cases, with this percentage being smaller for smaller sample sizes.

**Table 2:** Results for the unfolding focal item, displaying the percentage of replications in which support was found against monotonicity (outside of parentheses) and in favor of monotonicity (inside parentheses) for each of the seven measures, based on 100 replications.

*m*	$\alpha$	*R*	*N*	BF item level						BF category level							
				CP		CR		AC		CP		CR		AC		H	
2	0.75	5	200	76	(0)	62	(3)	58	(4)	61	(0)	62	(0)	59	(0)	100	(0)
			500	89	(0)	82	(0)	78	(0)	83	(0)	83	(0)	77	(0)	100	(0)
			1000	100	(0)	97	(0)	95	(0)	98	(0)	98	(0)	92	(0)	100	(0)
		10	200	86	(0)	76	(2)	72	(2)	76	(0)	78	(0)	77	(0)	100	(0)
			500	96	(0)	92	(0)	90	(1)	93	(0)	93	(0)	90	(0)	100	(0)
			1000	100	(0)	100	(0)	100	(0)	100	(0)	100	(0)	99	(0)	100	(0)
		20	200	96	(0)	80	(4)	78	(6)	93	(0)	92	(0)	92	(0)	100	(0)
			500	100	(0)	97	(0)	93	(1)	100	(0)	99	(0)	96	(0)	100	(0)
			1000	100	(0)	100	(0)	100	(0)	100	(0)	100	(0)	100	(0)	100	(0)
	1.5	5	200	92	(0)	88	(0)	85	(0)	87	(0)	83	(0)	81	(0)	99	(0)
			500	100	(0)	99	(0)	99	(0)	99	(0)	99	(0)	98	(0)	100	(0)
			1000	100	(0)	100	(0)	100	(0)	100	(0)	100	(0)	100	(0)	100	(0)
		10	200	99	(0)	97	(0)	94	(0)	97	(0)	98	(0)	98	(0)	100	(0)
			500	100	(0)	100	(0)	100	(0)	100	(0)	100	(0)	100	(0)	100	(0)
			1000	100	(0)	100	(0)	100	(0)	100	(0)	100	(0)	100	(0)	100	(0)
		20	200	100	(0)	100	(0)	97	(0)	100	(0)	100	(0)	100	(0)	100	(0)
			500	100	(0)	100	(0)	100	(0)	100	(0)	100	(0)	100	(0)	100	(0)
			1000	100	(0)	100	(0)	100	(0)	100	(0)	100	(0)	100	(0)	100	(0)
4	0.75	5	200	99	(0)	97	(0)	95	(1)	97	(0)	96	(0)	91	(0)	100	(0)
			500	100	(0)	100	(0)	100	(0)	100	(0)	100	(0)	99	(0)	100	(0)
			1000	100	(0)	100	(0)	100	(0)	100	(0)	100	(0)	100	(0)	100	(0)
		10	200	100	(0)	99	(0)	100	(0)	99	(0)	99	(0)	99	(0)	100	(0)
			500	100	(0)	100	(0)	100	(0)	100	(0)	100	(0)	100	(0)	100	(0)
			1000	100	(0)	100	(0)	100	(0)	100	(0)	100	(0)	100	(0)	100	(0)
		20	200	100	(0)	100	(0)	100	(0)	100	(0)	100	(0)	100	(0)	100	(0)
			500	100	(0)	100	(0)	100	(0)	100	(0)	100	(0)	100	(0)	100	(0)
			1000	100	(0)	100	(0)	100	(0)	100	(0)	100	(0)	100	(0)	100	(0)
	1.5	5	200	100	(0)	100	(0)	100	(0)	100	(0)	100	(0)	100	(0)	100	(0)
			500	100	(0)	100	(0)	100	(0)	100	(0)	100	(0)	100	(0)	100	(0)
			1000	100	(0)	100	(0)	100	(0)	100	(0)	100	(0)	100	(0)	100	(0)
		10	200	100	(0)	100	(0)	100	(0)	100	(0)	100	(0)	100	(0)	100	(0)
			500	100	(0)	100	(0)	100	(0)	100	(0)	100	(0)	100	(0)	100	(0)
			1000	100	(0)	100	(0)	100	(0)	100	(0)	100	(0)	100	(0)	100	(0)
		20	200	100	(0)	100	(0)	100	(0)	100	(0)	100	(0)	100	(0)	100	(0)
			500	100	(0)	100	(0)	100	(0)	100	(0)	100	(0)	100	(0)	100	(0)
			1000	100	(0)	100	(0)	100	(0)	100	(0)	100	(0)	100	(0)	100	(0)

Table 2 shows the results for the item for which the item scores were obtained using the unfolding model (i.e., the ‘unfolding item’). The item-level and category-level BF approaches generally result in comparable levels of power for detecting violations of monotonicity. For the five-category item, all conditions result in a very high probability of detecting the violations of monotonicity, exceeding 90% in all cases. For the three-category item, power is also adequate (exceeding 80% in all cases) when the manifest score is of high quality. When the manifest score is of lower quality, these power levels are achieved once a sample size of at least 500 is considered. The *H*-coefficient performs well for this item regardless of test length, quality, or sample size, which makes sense since one can expect low covariance between the score on the unfolding item and the scores on the other items, and hence one would expect *H*-values around 0 for this item.

As can be seen in Table 2 (numbers in parentheses), neither the category-level approach nor the *H*-coefficient resulted in replications where support for monotonicity was found for the unfolding item. For the item-level approach, there were a few conditions where in a small percentage of cases support was found for forms of monotonicity, which happened in at most 6% of cases and was mainly restricted to conditions with a small sample size. Overall, the false detection rates do not seem to be a problem for the item-level approach for this item.

**Table 3:** Results for the focal item with switched categories, displaying the percentage of replications in which support was found against monotonicity (outside of parentheses) and in favor of monotonicity (inside parentheses) for each of the seven measures, based on 100 replications.

*m*	$\alpha$	*R*	*N*	BF item level						BF category level							
				CP		CR		AC		CP		CR		AC		H	
2	0.75	5	200	9	(24)	89	(0)	72	(0)	39	(1)	99	(0)	99	(0)	95	(0)
			500	24	(18)	100	(0)	100	(0)	61	(0)	100	(0)	100	(0)	100	(0)
			1000	47	(3)	100	(0)	100	(0)	90	(0)	100	(0)	100	(0)	100	(0)
		10	200	14	(41)	86	(1)	79	(4)	62	(3)	100	(0)	100	(0)	99	(0)
			500	34	(32)	100	(0)	100	(0)	84	(1)	100	(0)	100	(0)	100	(0)
			1000	56	(12)	100	(0)	100	(0)	97	(0)	100	(0)	100	(0)	100	(0)
		20	200	16	(45)	90	(0)	81	(3)	82	(2)	100	(0)	100	(0)	100	(0)
			500	25	(44)	100	(0)	100	(0)	96	(0)	100	(0)	100	(0)	100	(0)
			1000	56	(22)	100	(0)	100	(0)	100	(0)	100	(0)	100	(0)	100	(0)
	1.5	5	200	30	(12)	99	(0)	96	(0)	59	(0)	100	(0)	100	(0)	36	(0)
			500	61	(1)	100	(0)	100	(0)	92	(0)	100	(0)	100	(0)	73	(0)
			1000	96	(1)	100	(0)	100	(0)	99	(0)	100	(0)	100	(0)	97	(0)
		10	200	26	(17)	100	(0)	97	(0)	89	(0)	100	(0)	100	(0)	39	(0)
			500	60	(6)	100	(0)	100	(0)	98	(0)	100	(0)	100	(0)	89	(0)
			1000	95	(0)	100	(0)	100	(0)	100	(0)	100	(0)	100	(0)	99	(0)
		20	200	41	(23)	98	(0)	96	(0)	96	(1)	100	(0)	100	(0)	41	(0)
			500	58	(17)	100	(0)	100	(0)	99	(0)	100	(0)	100	(0)	89	(0)
			1000	92	(1)	100	(0)	100	(0)	100	(0)	100	(0)	100	(0)	99	(0)
4	0.75	5	200	0	(100)	4	(80)	2	(93)	0	(56)	92	(0)	92	(0)	0	(98)
			500	0	(100)	12	(53)	14	(78)	1	(79)	100	(0)	100	(0)	0	(100)
			1000	0	(100)	67	(9)	49	(39)	0	(86)	100	(0)	100	(0)	0	(100)
		10	200	0	(100)	2	(93)	8	(91)	0	(74)	98	(0)	98	(0)	0	(100)
			500	0	(100)	24	(72)	41	(59)	0	(82)	100	(0)	100	(0)	0	(100)
			1000	0	(100)	65	(34)	78	(22)	0	(93)	100	(0)	100	(0)	0	(100)
		20	200	1	(99)	22	(78)	54	(46)	1	(81)	100	(0)	100	(0)	0	(99)
			500	2	(98)	60	(40)	84	(16)	0	(93)	100	(0)	100	(0)	0	(100)
			1000	1	(99)	83	(17)	98	(2)	1	(94)	100	(0)	100	(0)	0	(100)
	1.5	5	200	0	(100)	3	(72)	3	(94)	0	(49)	98	(0)	98	(0)	0	(100)
			500	0	(100)	30	(30)	36	(63)	0	(69)	100	(0)	100	(0)	0	(100)
			1000	0	(100)	90	(2)	85	(10)	0	(76)	100	(0)	100	(0)	0	(100)
		10	200	0	(100)	14	(84)	19	(81)	0	(70)	100	(0)	100	(0)	0	(100)
			500	0	(100)	52	(47)	69	(31)	0	(82)	100	(0)	100	(0)	0	(100)
			1000	1	(99)	92	(7)	97	(3)	1	(87)	100	(0)	100	(0)	0	(100)
		20	200	4	(96)	59	(41)	71	(29)	0	(78)	100	(0)	100	(0)	0	(100)
			500	6	(94)	77	(23)	96	(4)	2	(89)	100	(0)	100	(0)	0	(100)
			1000	8	(92)	99	(1)	100	(0)	3	(93)	100	(0)	100	(0)	0	(100)

The results for the item on which the scores were obtained by switching the last two categories (i.e., the ‘switched item’) are displayed in Table 3. There is a big discrepancy in the BF measures that focus on $\varvec{\xi }$ (cumulative probabilities) and those that focus on $\varvec{\phi }$ and $\varvec{\psi }$, with the former having a much lower power to detect a violation than the latter two. This is especially notable for the five-category item, where monotonicity of $\varvec{\xi }$ is only rarely rejected, regardless of sample size, test length, or quality of the manifest score, while the other two forms of monotonicity show a notable increase in rejection rates when increasing the sample size and when working with a manifest score of high rather than low quality. This notable difference between the detection rates of the three types of MM can be explained by considering the fact that the nonmonotonicity of $\varvec{\xi }$ that is present at the latent level is located at the high-end of the ability scale, such that it does not or only very slightly show(s) up as a decrease in the manifest cumulative probabilities (depending on the test length and on the number of categories). Consequently, for some tests (five-category items, $K = 5$ or 10) this type of violation of latent monotonicity of the cumulative probabilities may simply not be detectable at all, regardless of the considered sample size. This problem does not hold for the other two types of monotonicity, where the induced violation at the latent level always translates to a notable violation of MM, and detecting this violation is just a matter of having a sufficiently large sample size.

When comparing the performance of the item-level approach with that of the category-level approach, the latter quite strongly outperforms the former. By considering the sets of constraints for each particular category separately rather than considering the item as a whole, the category-level approach is more easily able to detect the problems present for the highest two categories. The item-level approach likely suffers in power due to the lower categories being ‘well behaved,’ such that the positive support found for those categories may to some degree mask the problems with the last two categories. This is especially problematic in conditions considering the five-category item, since the item-level approach often suggests there to be support in favor of monotonicity rather than against it, resulting in a large proportion of incorrect conclusions. While this percentage decreases notably with sample size, this does suggest that for smaller sample sizes the item-level approach may allow negative evidence relating to some of the categories to be overwhelmed by positive evidence relating to other categories, a problem that does not occur for the category-level approach.

For the *H*-coefficient, the outcome seems to depend heavily on whether a three- or a five-category item is considered. For the three-category item support is never found, and for the larger sample sizes it is generally concluded that the item is not well behaved. In contrast, for the five-category item it is almost always concluded that the item behaves well, and hence the item would not get flagged.

## Empirical Example

The methods for testing latent monotonicity were applied to a data set with responses to the Radical Feminist Perspective scale, which is a scale for measuring feminist and gender attitudes (Henley, Meng, O’Brien, McCarthy, & Sockloskie, 1998). The scale consists of 10 items, each presenting the respondent with a statement to which they can rate their degree of agreement using one of five response categories (‘disagree,’ ‘slightly disagree,’ ‘neutral,’ ‘slightly agree,’ ‘agree’). All items in the scale were formulated such that agreement was meant to be indicative of higher levels of the attitude. Responses of 1000 persons on the 10 items were randomly selected from a larger data set.

To assess whether monotonicity holds for the items in the scale, the three forms of monotonicity were evaluated both on the item level and the category level. In line with the recommendations made earlier in the paper, as the manifest score the restscore based on the dichotomized items scores (based on a median split) was used. This choice was made with the explicit purpose of obtaining a manifest score for which the assumption of stochastic ordering of the latent variable may be considered plausible. While SOL can generally be considered plausible for scales that consist mostly of well-behaved dichotomous (or dichotomized) items, it should be noted that this property is not guaranteed by the procedure and that in principle other manifest scores can be considered as well if those are deemed to provide a better proxy for the latent variable. BFs were estimated using 100,000 burn-in and at least 100,000 burn-in iterations, allowing for up to 50 million iterations if the fit of a constraint would initially be estimated at 0.

**Table 4:** Item-level and category-level monotonicity results for the Radical Feminist Perspective scale.

*i*	CP					CR					AC				
	Item	C1	C2	C3	C4	Item	C1	C2	C3	C4	Item	C1	C2	C3	C4
1	16.36	8.28	9.71	7.43	5.32	8.68	8.28	7.10	$-$ 0.71	$-$ 5.66	12.55	2.74	5.07	$-$ 1.69	$-$ 5.66
2	28.61	11.09	10.18	12.06	12.30	37.46	11.09	8.00	9.37	8.95	37.97	4.87	2.70	5.14	8.95
3	30.25	8.99	11.16	12.89	14.31	43.06	8.99	10.49	11.54	12.11	38.52	2.17	4.31	8.29	12.11
4	31.59	11.93	12.35	13.83	12.37	40.03	11.93	9.63	10.99	7.51	44.81	3.90	6.11	10.85	7.51
5	31.15	11.50	12.82	13.49	11.98	35.25	11.50	11.47	9.94	2.59	40.56	8.14	4.63	6.83	2.59
6	32.48	11.77	12.23	12.53	12.50	41.58	11.77	10.16	9.11	10.57	38.35	5.09	3.08	4.43	10.57
7	31.43	10.53	12.41	13.76	12.76	43.24	10.53	12.13	11.58	9.08	42.62	4.73	6.34	8.79	9.08
8	29.58	13.79	12.88	11.39	9.71	23.18	13.79	9.14	0.06	0.36	31.20	9.51	5.34	1.10	0.36
9	20.12	9.60	10.85	8.60	5.47	17.11	9.60	6.65	0.93	$-$ 0.19	23.88	6.39	3.47	1.02	$-$ 0.19
10	30.03	9.79	11.79	10.33	12.42	34.29	9.79	8.43	5.87	10.05	16.32	$-$ 11.31	2.84	1.74	10.05

Here, *i* refers to the item number, and CP, CR, and AC stand for monotonicity of the cumulative probability, continuation ratio, and adjacent categories building blocks, respectively. *Item* shows $\ln ({\hbox {BF}})$ for the item-level $\ln ({\hbox {BF}})$, *C1* for the first set of constraints, *C2* for the second set of constraints, *C3* for the third set of constraints, and *C4* for the fourth set of constraints of each item.

The results of the analysis are presented in Table 4. When considering the item-level results, all ten items show support for all three forms of monotonicity ($\ln ({\hbox {BF}}) > 3$). While these results seem to suggest that the items are all well behaved, the results of the simulation study indicated that item-level results may obscure problems with specific categories and hence that critically considering the category-level results is important as well. For the cumulative probabilities, all category-level results suggest support in favor of monotonicity (i.e., $\ln ({\hbox {BF}}) > 3$ for all four sets of constraints). For the continuation ratios, support was found for monotonicity for six of the items, while the results were inconclusive for three items (item 5, 6, and 7), and evidence against monotonicity was found for one item (item 1). For the adjacent categories, support in favor of monotonicity was found for only three items (item 4, 6, and 7), while evidence against monotonicity was found for item 1 and item 10.

For the items where evidence against some form of monotonicity was found at the category level, these issues seem to concern the extreme categories: For item 1, the ordering of the two highest categories seems problematic, as the probability of choosing ‘agree’ over ‘slightly agree’ does not appear to increase monotonically over the manifest score ($\ln ({\hbox {BF}}) = -5.66$), while for item 10 the probability of choosing ‘slightly disagree’ over ‘disagree’ does not appear to increase monotonically ($\ln ({\hbox {BF}}) = -11.31$). This suggests that these categories may not function well for these particular items, a result that would have been masked if only the item-level results would have been considered. For these two items, merging the problematic categories before continuing with subsequent IRT analyses might be considered if one decides not to discard the items altogether.

## Discussion

Two approaches to evaluating the three types of latent monotonicity were considered: summarizing the evidence at the item level versus at the category level. Using an item-level measure to evaluate monotonicity has the elegance of providing one overall measure for each form of monotonicity. The simulation study also shows that such an approach more easily finds support in favor of monotonicity for well-functioning items since all the evidence is combined. However, by combining all the evidence and only considering this at the item level, one can also more easily be misled if there only is a problem with a few of the categories, while the other categories function normally, as appeared to be the case in the empirical example. The simulation study showed that this can result in a false positive rate that exceeds acceptable limits. As the situation where problems are due only to a subset of the categories is exactly the kind of situation that might be easily overlooked in practice and hence where one would hope that procedures such as these provide added value, this disadvantage may motivate one to consider using sets of category-level measures instead.

The category-level approach as it was proposed here is more strict than the item-level approach: Only if for each category support is found in favor of monotonicity does one conclude that monotonicity is supported, and vice versa one already concludes that there is evidence against monotonicity if one of the ln(BF)s falls below $-3$, even if the item-level BF would have indicated support for monotonicity (which was the case for two items in the empirical example). The implication of this is that the category-level approach is somewhat less powerful in finding evidence in favor of monotonicity for well-functioning items, especially when items use many categories, since the number of considered BFs is equal to *m*. However, this loss of power to detect support for MM is likely outweighed by the improvement in the false positive rates compared to the item-level approach, since false positives are rare when using the category-level approach, especially when a reasonable sample size is considered. Based on the simulation study, the power to detect violations of monotonicity when they are present seems to be at least comparable and sometimes much better than that of the item-level approach. It also allows one to specifically consider the functioning of individual categories, which provides fine-grained relevant information about the functioning of these categories for each item, as was illustrated in the empirical example. This together suggests that overall the use of the category-level approach is generally to be preferred over the item-level approach.

Both approaches make use of BFs to evaluate the assumption that latent monotonicity holds. While model assumptions are commonly evaluated using significance tests, using BFs allows one to better answer the question that is central when evaluating model assumptions, namely whether it is plausible that the assumption holds. An important benefit of BF methods is that they allow for quantifying positive support, and hence give users tools for determining whether they can be confident about the functioning of the items in their test. While with the proposed procedure inconclusive results can be obtained (if $-3< \ln ({\hbox {BF}}) < 3$), these Bayesian methods make it possible to continue gathering data until one reaches the point where conclusive results have been obtained (Rouder, 2014). Additionally, the choice of the BF-thresholds that are used to evaluate monotonicity can be adapted based on the needs of the user: More conservative or liberal bounds can be used depending on the amount of support one would like to see before concluding that monotonicity is supported or should be rejected for an item, for example if one is concerned about multiple testing. Kass & Raftery  (1995) provide relevant guidelines for interpreting the amount of support indicated by specific BF values.

The proof provided in this paper shows that if one considers a manifest score for which the property of SOL holds, each form of latent monotonicity translates into a form of MM. Consequently, the procedures developed in this paper work under the assumption that monotonicity is evaluated using a manifest score for which SOL holds. As was indicated, this assumption is not automatically warranted, and the selection of the manifest score should be done with care. Because standard IRT models for dichotomous item responses all imply SOL for the restscore while most polytomous IRT models do not, our general recommendation has been to use a manifest score based on dichotomized item scores rather than polytomous item scores. While this may make it more plausible that SOL holds for the considered restscore, the restscore can still be ‘contaminated’ if for a notable proportion of the dichotomized items latent monotonicity is violated. Hence, the selection of the manifest score should be done with care, and eliminating items that are not well behaved from that manifest score is advisable.

As indicated, all common parametric IRT models for polytomous data assume the three forms of latent monotonicity considered in this paper. As the three forms of monotonicity are nested, one could argue that only monotonicity of the adjacent-category ratios needs to be considered. However, from a measurement perspective the three forms of monotonicity focus on different properties of the item, making it relevant to consider each in their own right. Furthermore, if one considers using nonparametric polytomous IRT models, these tests can help one decide which model might be appropriate. Additionally, considering all three forms of latent monotonicity may be helpful to determine the nature of a possible violation.

### Electronic supplementary material

Below is the link to the electronic supplementary material.

Supplementary material 1 (R 13 KB)

Supplementary material 2 (c 42 KB)

## Appendix A: Lower and Upper Bounds for $\varvec{\phi }_{ij}$

(1) **Adjacent-category ratios**

Since $\psi _{(j-1)r}$ and $\psi _{jr}$ are functions of $\phi _{jr}$, $\phi _{jr}$ is included in the following constraints for the adjacent-category ratios:

33 $$\begin{aligned} \psi _{jr}&=\frac{\phi _{jr}(1-\phi _{(j+1)r})}{1-\phi _{jr}\phi _{(j+1)r}}\ge&\psi _{j(r-1)},&\forall j\in [1:m],&\forall r=[1:s]; \end{aligned}$$

34 $$\begin{aligned} \psi _{jr}&=\frac{\phi _{jr}(1-\phi _{(j+1)r})}{1-\phi _{jr}\phi _{(j+1)r}}\le&\psi _{j(r+1)},&\forall j\in [1:m],&\forall r=[0:(s-1)];\end{aligned}$$

35 $$\begin{aligned} \psi _{(j-1)r}&=\frac{\phi _{(j-1)r}(1-\phi _{jr})}{1-\phi _{(j-1)r}\phi _{jr}}\ge&\psi _{(j-1)(r-1)},&\forall j\in [2:m],&\forall r=[1:s];\end{aligned}$$

36 $$\begin{aligned} \psi _{(j-1)r}&=\frac{\phi _{(j-1)r}(1-\phi _{jr})}{1-\phi _{(j-1)r}\phi _{jr}}\le&\psi _{(j-1)(r+1)},&\forall j\in [2:m],&\forall r=[1:(s-1)]. \end{aligned}$$

Each of these constraints is a linear constraint on $\phi _{jr}$, and the following lower ($a_{\psi jrs}$) and upper bounds ($b_{\psi jrs}$) can be derived from them:

37 $$\begin{aligned} a_{\psi jrs}= & {} {\left\{ \begin{array}{ll}0,\text { if }j=1,r=0;\\ \frac{\phi _{(j-1)r}-\psi _{(j-1)(r+1)}}{\phi _{(j-1)r}\left( 1-\psi _{(j-1)(r+1)}\right) },\text { if }{j=1,r\in [1:s],}\text { and }j\in [2:m],r=0;\\ \max \left( \frac{\phi _{(j-1)r}-\psi _{(j-1)(r+1)}}{\phi _{(j-1)r}\left( 1-\psi _{(j-1)(r+1)}\right) }, \frac{\psi _{j(r-1)}}{1+\phi _{(j+1)r}\left( 1-\psi _{j(r-1)}\right) } \right) ,\text { if }j\in [2:m],r\in [1:(s-1)];\\ \frac{\psi _{j(r-1)}}{1+\phi _{(j+1)r}\left( 1-\psi _{j(r-1)}\right) },\text { if }j\in [2:m],r=s. \end{array}\right. }\nonumber \\ \end{aligned}$$

38 $$\begin{aligned} b_{\psi jrs}= & {} {\left\{ \begin{array}{ll} \frac{\psi _{j(r+1)}}{1+\phi _{(j+1)r}\left( 1-\psi _{j(r+1)}\right) },\text { if }j=1,r\in [0:(s-1)],\text { and }j\in [2:m],r=0;\\ \min \left( \frac{\phi _{(j-1)r}-\psi _{(j-1)(r-1)}}{\phi _{(j-1)r}\left( 1-\psi _{(j-1)(r-1)}\right) }, \frac{\psi _{j(r+1)}}{1+\phi _{(j+1)r}\left( 1-\psi _{j(r+1)}\right) }\right) ,\text { if }j\in [2:m],r\in [1:(s-1)];\\ 1,\text { if }j=1,r=s;\\ \frac{\phi _{(j-1)r}-\psi _{(j-1)(r-1)}}{\phi _{(j-1)r}\left( 1-\psi _{(j-1)(r-1)}\right) },\text { if }j\in [2:m],r=s. \end{array}\right. }\nonumber \\ \end{aligned}$$

(2) **Continuation ratios**

The constraints on $\phi _{jr}$ do not require additional transformation, and hence the lower ($a_{\phi jrs}$) and upper bounds ($b_{\phi jrs}$) are

39 $$\begin{aligned} \forall j\in [1:m], a_{\phi jrs}= & {} {\left\{ \begin{array}{ll} 0,\text { if }r=0;\\ \phi _{j(r-1)},\text { if }r\in [1:s]; \end{array}\right. } \end{aligned}$$

40 $$\begin{aligned} \forall j\in [1:m], b_{\phi jrs}= & {} {\left\{ \begin{array}{ll} \phi _{j(r+1)},\text { if }r\in [0:(s-1)];\\ 1,\text { if }r=s.\\ \end{array}\right. } \end{aligned}$$

(3) **Cumulative probabilities**

Since $\xi _{kr}$ is a function of $\phi _{jr},\forall j\in [1:m], k\in [j:m]$, $\phi _{jr}$ is included in the following constraints:

41 $$\begin{aligned} \xi _{kr}&=\phi _{jr}\prod _{v=1,v\ne j}^k \phi _{vr}\ge \xi _{k(r-1)}, \forall r\in [1:s]; \end{aligned}$$

42 $$\begin{aligned} \xi _{kr}&=\phi _{jr}\prod _{v=1,v\ne j}^k \phi _{vr}\le \xi _{k(r+1)}, \forall r\in [0:(s-1)], \end{aligned}$$

for all $k\in [j:m]$. Each of these constraints is linear in $\phi _{jr}$ and to satisfy all of them, $\phi _{jr}$ needs to be no smaller than the lower bound $a_{\xi jrs}$ and no larger than the upper bound $b_{\xi jrs}$, which are:

43 $$\begin{aligned} \forall j\in [1:m], a_{\xi jrs}= & {} {\left\{ \begin{array}{ll} 0,\text { if }r=0;\\ \max \limits _{k\in [j:m]}\left( \frac{\xi _{k(r-1)}}{\prod \limits _{v=1,v\ne j}^k\phi _{vr}}\right) ,\text { if }r\in [1:s]; \end{array}\right. } \end{aligned}$$

44 $$\begin{aligned} \forall j\in [1:m], b_{\xi jrs}= & {} {\left\{ \begin{array}{ll} \min \limits _{k\in [j:m]}\left( \frac{\xi _{k(r+1)}}{\prod \limits _{v=1,v\ne j}^k\phi _{vr}}\right) ,\text { if }r\in [0:(s-1)];\\ 1,\text { if }r=s. \end{array}\right. } \end{aligned}$$

## Appendix B: Gibbs Sampler for the Item-Level Bayes Factors

Here, we describe the steps of the Gibbs sampler for estimating $g(Q_{z(s+1)}\,|\, Q_{z1},\ldots ,Q_{zs})$:

*Steps from* 1 *to**s* For each $r\in [0:s]$, consecutively sample the elements of $\varvec{\phi }_{\varvec{\cdot } r}$ from truncated Beta distributions:

45 $$\begin{aligned} \forall j\in [1:m], \phi _{jr}\sim \mathcal {B}\left( m-j+1+\sum _{k=j}^mN_{kr},1+N_{(j-1)r}\right) \mathcal {I}(a_{zjrs}\le \phi _{jr}\le b_{zjrs}). \end{aligned}$$

If $N_{(j-1)r}=0$, one can sample from (45) using the CDF inversion technique since the posterior is in that cases proportional to $\phi _{jr}^{m-j+\sum _{k=j}^mN_{kr}}$. When $N_{jr}>0$, we use the data augmentation scheme of Damien and Walker (2001) to sample from the truncated Beta distribution in (45), which entails introducing an auxiliary variable $v_{jr}$ and sampling from the joint distribution

46 $$\begin{aligned} f(\phi _{jr},v_{jr})\propto \phi _{jr}^{m-j+\sum _{k=j}^mN_{kr}}\mathcal {I}(0\le v_{jr}\le (1-\phi _{jr})^{N_{(j-1)r}}) \end{aligned}$$

in two steps: (a) Sample the auxiliary variable $v_{js}$ from $\mathcal {U}(0,(1-\phi _{jr})^{N_{(j-1)r}})$; (b) sample $\phi _{js}$ from

47 $$\begin{aligned} \phi _{jr}^{m-j+\sum _{k=1}^mN_{kr}} \mathcal {I}\left( a_{zjrs}\le \phi _{jr}\le \min \left( b_{zjrs},1-v_{jr}^{\frac{1}{N_{(j-1)r}}}\right) \right) \end{aligned}$$

using the CDF inversion technique.

*Step*$s+1$ Sample the elements of $\varvec{\phi }_{\varvec{\cdot } (s+1)}$ from their unconstrained Beta distributions:

48 $$\begin{aligned} \forall j\in [1:m], \phi _{j(s+1)}\sim \mathcal {B}\left( 1+m-j+\sum _{k=j}^mN_{k(s+1)},1+N_{(j-1)(s+1)}\right) . \end{aligned}$$

## Appendix C: Gibbs Sampler for the Category-Level Bayes Factors

The Gibbs sampler for sampling from the

49 $$\begin{aligned} f(z_{j0},\ldots ,z_{j(s+1)}\,|\, \mathbf {X},Q_{zj1},\ldots ,Q_{zjs}) \end{aligned}$$

has the following steps:

*Step 1* Sequentially sample $z_{j0},\ldots ,z_{js}$ from their conditional posteriors which are constrained Beta distributions:

50 $$\begin{aligned}&z_{j0}\sim \mathcal {B}(N_{zj0},S_{zj0})\mathcal {I}(z_{j0}\le z_{j1}), \end{aligned}$$

51 $$\begin{aligned}&\forall r\in [1:(s-1)]: z_{jr}\sim \mathcal {B}(N_{zjr},S_{zjr})\mathcal {I}(z_{j(r-1)}\le z_{jr}\le z_{j(r+1)}), \end{aligned}$$

52 $$\begin{aligned}&z_{js}\sim \mathcal {B}(N_{zjs},S_{zjs})\mathcal {I}(z_{j(s-1)}\le z_{js}), \end{aligned}$$

sampling from which can be done using a data augmentation algorithm as explained in “Appendix B.”

*Step 2* Sample $z_{j(s+1)}$ from $\mathcal {B}(z_{j(s+1)};N_{zj(s+1)},S_{zj(s+1)})$.

## Footnotes

1 It may be noted that in the literature this is often simply called ‘latent monotonicity’ for polytomous response data (e.g., see Holland & Rosenbaum, 1985), but as there are other forms of latent monotonicity that also play a role in polytomous IRT, in this paper the term is not used exclusively for monotonicity of $\xi _{ij}(\theta )$.

2 Here, it may be noted that since a BF can indicate inconclusive evidence, concluding that the false negative and false positive rate are low does not guarantee a high true positive and true negative rate, and hence all four need to be considered.

3 It may be noted that the $\phi _{(m+1)r}$s and $\xi _{(m+1)r}$s are only considered for notational convenience and are not part of the matrices $\varvec{\phi }$ and $\varvec{\xi }$.
